# The Heparin-Binding Activity of Secreted Modular Calcium-Binding Protein 1 (SMOC-1) Modulates Its Cell Adhesion Properties

**DOI:** 10.1371/journal.pone.0056839

**Published:** 2013-02-21

**Authors:** Marina Klemenčič, Marko Novinec, Silke Maier, Ursula Hartmann, Brigita Lenarčič

**Affiliations:** 1 Department of Chemistry and Biochemistry, Faculty of Chemistry and Chemical Technology, University of Ljubljana, Ljubljana, Slovenia; 2 Center for Biochemistry, Medical Faculty, University of Cologne, Cologne, Germany; 3 Cologne Excellence Cluster on Cellular Stress Responses in Aging-Associated Diseases, Cologne, Germany; 4 Department of Biochemistry, Molecular and Structural Biology, Jožef Stefan Institute, Ljubljana, Slovenia; University of Patras, Greece

## Abstract

Secreted modular calcium-binding proteins 1 and 2 (SMOC-1 and SMOC-1) are extracellular calcium- binding proteins belonging to the BM-40 family of proteins. In this work we have identified a highly basic region in the extracellular calcium-binding (EC) domain of the SMOC-1 similar to other known glycosaminoglycan-binding motifs. Size-exclusion chromatography shows that full length SMOC-1 as well as its C-terminal EC domain alone bind heparin and heparan sulfate, but not the related chondroitin sulfate or dermatan sulfate glycosaminoglycans. Intrinsic tryptophan fluorescence measurements were used to quantify the binding of heparin to full length SMOC-1 and the EC domain alone. The calculated equilibrium dissociation constants were in the lower micromolar range. The binding site consists of two antiparallel alpha helices and mutagenesis experiments have shown that heparin-binding residues in both helices must be replaced in order to abolish heparin binding. Furthermore, we show that the SMOC-1 EC domain, like the SMOC-2 EC domain, supports the adhesion of epithelial HaCaT cells. Heparin-binding impaired mutants failed to support S1EC-mediated cell adhesion and together with the observation that S1EC in complex with soluble heparin attenuated cell adhesion we conclude that a functional and accessible S1EC heparin-binding site mediates adhesion of epithelial cells to SMOC-1.

## Introduction

Both secreted modular calcium-binding proteins 1 and 2 (SMOC-1 and SMOC-2) are involved in direct or indirect modulation of growth factor signaling pathways and play diverse roles in physiological processes involving extensive tissue remodeling. Namely, it has been shown that SMOC-1 acts as a regulator of osteoblast differentiation [1] and is involved in inhibition of transforming growth factor-β signaling through production of nitric oxide [2]. Recently, three unrelated papers reported on SMOC-1 being essential for ocular and limb development and two point mutations in the *SMOC1* gene were shown to cause Waardenburg Anophtalmia Syndrome [3], [4], [5]. On the other hand, SMOC-2 enhanced proliferative response to basic fibroblast growth factor (bFGF) and stimulated DNA synthesis in cultured human umbilical vein endothelial cells via interaction with vascular endothelial growth factor or bFGF [6]. Furthermore, ectopically expressed SMOC-2 stimulated the formation of network-like structures in an *in vitro* matrigel angiogenesis assay [7]. The SMOCs are members of the BM-40 family, which comprises proteins that contain a follistatin-like domain (FS) and an extracellular calcium-binding (EC) domain with two EF hands. In most members of the family the FS and EC domains occur in tandem, in the SMOCs, however, they are separated by two thyroglobulin type-1 domains and a domain, unique to SMOC proteins [8], [9].

The EC domain of BM-40 has been shown to stimulate cell adhesion [10], inhibit proliferation and abrogate focal adhesions [11]. A similar effect was reported for hevin, another member of BM-40 family, which inhibited adhesion and spreading of endothelial cells [12]. In contrast, the EC domain of SMOC-2 stimulated migration as well as adhesion of keratinocyte-like HaCaT cells. The latter property was attributed to an interaction of the EC domain with integrins αvβ1 and αvβ6 [13] which are important players in cell–cell and cell–matrix adhesion processes. αvβ1 is a fibronectin receptor [14], while αvβ6 integrin was shown to bind to numerous proteins such as fibronectin, vitronectin, tenascin-C and osteopontin [15]. Interestingly, vitronectin has also been shown to bind both SMOCs *in vitro* [16]. Increasing evidence now indicates that integrin-mediated adhesion can depend on the co-receptor function of heparan sulfate (HS) proteoglycans (HSPGs) [17]. HSPGs are extracellular matrix (ECM) proteins they have been receiving increasing attention due to the growing evidence of the important roles they play in regulation of physiological processes. They consist of a protein core to which one or more HS glycosaminoglycan (GAG) chains are covalently bound [18]. HS is produced by the vast majority of cells. It is a linear polymer composed of repeating disaccharide units consisting of α-D-glucosamine and iduronic or glucuronic acid. HS chains can vary in length and degree of sulfation (usually 0.6–1.5 sulfates/disaccharide) [19]. A special type of HS is heparin (HP), a highly sulfated polymer (2.3–2.8 sulfates/disaccharide) and known anticoagulant, produced exclusively by mast cells. HSPGs are main constituents of basement membranes (BMs) [20], thin structures that separate endothelial or epithelial cells from subjacent tissues. Interestingly, SMOC-1 was shown to localize predominantly to BMs [8].

In this paper we identify SMOC-1, and by implication SMOC-2, as HS-binding proteins and map the HS-binding site to the C-terminal EC domain. We characterize the interaction *in vitro* and show that the SMOC-1 EC domain supports epithelial HaCaT cell adhesion via a HS-dependent mechanism. These data advance our knowledge on the physiological role of SMOC-1 and its EC domain in epithelial cell adhesion.

## Materials and Methods

### Materials

The cDNA coding for full-length SMOC-1 (S1FL) was obtained from RZPD, Germany (clone IDs IRAKp961G1312Q2). DNA primers, PCR enzyme selection kit and Penstrep were from Invitrogen (USA). The pET-28b(+) vector and *Escherichia coli* strain BL21(DE3) were from Novagen (Germany). The human epithelial HaCaT cell line was obtained from ATCC (American Type Culture Collection) [21]. The *XhoI* and *NcoI* restriction enzymes were from Fermentas, Thermo Scientific (USA). The Low Molecular Weight Calibration Kit, Chelating Sepharose Fast Flow, Heparin Sepharose 6 Fast Flow, the Superdex 200 column and the ÄKTA FPLC system were from GE Healthcare (Sweden) and the Q or S columns were from BioRad (USA). Dulbecco’s Modified Eagle Medium (DMEM), RPMI-1640, Dulbeccòs Phosphate Buffered Saline (DPBS) and fetal bovine serum (FBS) were from Gibco (USA). Dalton Standards MS-II were from Serva (Germany). All cell cultureware were from BD Biosciences (USA). The Cy-3 labeled anti-mouse IgG was from Jackson ImmunoResearch (USA) and the Alexa 488-labeled goat anti-rabbit IgG from Molecular Probes (USA). The β6 integrin was kindly provided by Dean Sheppard, San Francisco. Adhesion assays were performed in Clear 96-well Microtest™ Plates and spreading assays on Lab-Tek® Chamber slides from Nunc (USA). The rabbit anti-vinculin antibody, bovine serum albumin (BSA), heparin sodium salt (HP), dermatan sulfate (DS), chondroitin sulfate (CS), sodium chlorate and human plasma fibronectin were from Sigma-Aldrich (USA). Heparan sulfate (HS) was from Iduron (UK). The weight average molecular masses, *M_w_*, for GAGs were 15 kDa for HP and HS, 23 kDa for CS and 26 kDa for DS. All data processing and figures were prepared using GraphPad Prism 5.0 software.

### Cloning and Expression of Recombinant Proteins

Recombinant murine S1EC was cloned, expressed and purified as described previously [13] for SMOC-2 EC domain using primers described in Supporting Methods S1. Coding sequences for human S1FL, S1EC and three S1EC mutants (MUTE, MUTF and DBMUT) were amplified by PCR (see Supporting Methods S1 for a detailed description) and cloned into the bacterial expression vector pET-28b(+) in frame with a C-terminal hexahistidine tag using *NcoI* and *XhoI* restriction sites. Correct ligation and in-frame insertion of the fragments were verified by DNA sequencing. *Escherichia coli* strain BL21(DE3) bacteria were transformed with the expression plasmids and grown in shaker cultures at 37°C in LB medium containing 30 µg/ml kanamycin. When the cell density reached an OD_600_ of 0.8, expression of the recombinant proteins was induced by addition of IPTG to a final concentration of 1 mM. After induction, cells were grown for an additional 3 h and harvested by centrifugation at 5000 g for 10 min.

### Purification of Recombinant Proteins

Expressed proteins were isolated from inclusion bodies, purified and refolded as described previously [16]. Recombinant SMOC-1 and S1EC were dialyzed against buffer H (20 mM HEPES pH 7.4, 150 mM NaCl, 2 mM CaCl_2_) and applied to a heparin sepharose column equilibrated in the same buffer. The column was thoroughly washed with binding buffer and bound proteins were eluted with 1 M NaCl in buffer H. MUTE and MUTF were dialyzed against buffer C (20 mM acetate, pH 6.5, 2 mM CaCl_2_) and applied to a S-cation exchange column, pre-equilibrated in the same buffer. Bound proteins were washed with buffer C and eluted in a linear gradient from 0 to 1 M NaCl. MUTE and MUTF were further purified by heparin affinity chromatography as described for SMOC-1 and S1EC. DBMUT was dialyzed against buffer D (20 mM HEPES, pH 8.0, 2 mM CaCl_2_) and applied to a Q-anion exchange column. After an extensive wash with buffer D, bound proteins were eluted in a linear gradient from 0 to 1 M NaCl. The purity of all isolated proteins was assessed by SDS-PAGE (Supporting Figure S1). All proteins were dialyzed against buffer H, aliquoted and stored at −80°C.

### Analysis of GAG Binding by SEC

Purified proteins (final concentration of 1 mg/ml) were incubated with HP, HS, CS or DS (1 mg/ml final concentration) and applied to a Superdex 200 size-exclusion chromatography (SEC) column connected to ÄKTA FPLC system. The column was equilibrated in 20 mM HEPES pH 7.4, 150 mM NaCl, 2 mM CaCl_2_. Elution diagrams were recorded and compared to control samples containing recombinant protein alone. A sample of Dalton MS-II standard proteins was run in parallel for calibration.

### Molecular Modeling of S1EC

The homology model of S1EC was built using Modeller 9v10 software. The crystal structure of the EC domain of BM-40 (PDB ID 1SRA) was used as the template for modeling. The sequence alignment was calculated using the PROMALS web server [22] and residues 381 through 392 were restricted to an alpha-helical conformation. Ten models were calculated and the best model was selected based on the values of all available scoring functions (molpdf, GA433, DOPE and DOPE-HR). All images were prepared using PyMOL (DeLano Scientific, Ltd.).

### Heparin Docking

Coordinates of heparin octasaccharides were extracted from the solution structure of heparin (PDB ID 1HPN) separately for each of the two available conformations (iduronic acid in the 1C4 and 2S0 conformation, respectively). The octasaccharides were docked to the S1EC domain using Autodock 4.2. The region of alpha helices E and F was defined as the binding site using AutoGrid 4.2 and the octasaccharides docked to the receptor using the Lamarckian Genetic Docking Algorithm. The glycosidic bonds in the ligands were defined as rigid, while the sulfate and carboxylate groups were defined as flexible. The best docking solution was selected based on the calculated binding constant and taking into account the experimentally determined restraints.

### Intrinsic Tryptophan Fluorescence and Determination of the Dissociation Constant *K*_d_

The interaction of recombinant proteins with heparin was monitored by measuring the intrinsic tryptophan fluorescence using a PerkinElmer LS 50B spectrofluorimeter. The excitation wavelength at 295 nm was used to avoid excitation of tyrosine or phenylalanine residues. Emission spectra were recorded 5 times over the range of 300 to 500 nm at 100 nm/min and excitation and emission slit widths of 10 nm. All measurements were performed using a 5 µM final protein concentration in buffer H at 25°C in a 1×1 cm quartz cuvette. Increasing amounts of heparin were added to the reaction mixture. In all experiments the total change of reaction volume due to addition of heparin was less than 2%. Where denoted, EDTA was added to a final concentration of 5 mM prior to addition of heparin. Emission spectra of the buffer in the presence or absence of heparin were recorded separately and were subtracted from the protein emission spectra.

To quantify the interaction between SMOC-1 and heparin, the intensities of fluorescence at a fixed wavelength (355 nm for SMOC-1 and 338 nm for S1EC) were measured. The degree of saturation (F_a_) was determined by transforming the experimental data to the form:
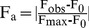
(1)where F_0_ is the fluorescence intensity in the absence of heparin, F_obs_ is the fluorescence intensity in the presence of non-saturating concentrations of heparin and F_max_ is the fluorescence intensity at saturation. The data was then plotted in the form of F_a_ versus heparin concentration and analyzed by non-linear regression using the equation for tight binding of ligand [23]:
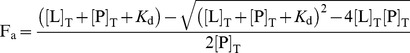
(2)where 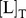 and 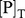 are the total concentrations of heparin and protein in the reaction mixture and 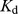 is the equilibrium dissociation constant of the complex. To calculate the value of 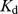, Equation 2 was fitted to the experimental data using GraphPad Prism 5.0 software.

### CD Measurements

CD spectra were collected on an Aviv 62DS spectropolarimeter (Aviv Inc.), using a 0.2 mm path-length quartz cuvette and averaging three repetitive scans between 260 and 190 nm. Spectra were recorded using 5 µM final protein concentration at 25°C in buffer H. The conformational changes were tested by addition of 4 mM EDTA or 100 µM heparin. The molar ellipticity [θ] was calculated using the calculated relative molecular masses of the recombinant protein.

### Cell Culture

Immortalized human HaCaT keratinocytes were cultured in Dulbecco’s modified Eagle’s (DMEM) medium supplemented with 10% fetal bovine serum (FBS) and the antibiotics penicillin (1000 units/ml) and streptomycin (1 mg/ml). Cells were maintained in a humidified atmosphere of 95% air and 5% carbon dioxide at 37°C.

### Adhesion Assay

Microtiter plates were coated with increasing amounts of recombinant proteins (0–40 µg/ml) in PBS overnight at 4°C. Human plasma fibronectin served as a positive control and wells without any coated protein as a negative control. Wells were blocked for 4 h at 4°C with 100 µl 1% (w/v) BSA in PBS. Where indicated, EGTA or heparin was mixed with the cells prior to plating. HaCaT cells were trypsinized, resuspended in serum-free medium at a cell density of 1×10^6^ cells/ml and 100 µl of the cell suspension were added to the wells. Cells were incubated for 30 min at 37°C. Non-adherent cells were removed by three PBS washes, and adherent cells were fixed in 4% formaldehyde. The cells were then washed and stained with 0.1% cresyl violet acetate for 30 min. Followed by a thorough wash, a solubilisation solution of 10% acetic acid was added to the wells. The colored solution was quantified using a Tecan Sunrise 96-well plate reader at 590 nm. Measurements were done in triplicates and results calculated as mean in each experiment. Inhibition of GAG sulfation [24] was achieved by growing the cells at least 24 h before adhesion assay in RPMI-1640 medium supplemented with 10% FBS dialyzed against PBS and containing the indicated amount of sodium chlorate. Cells were then detached and plated for cell adhesion assay as described above.

### Cell Spreading

Lab-Tek® Chamber slides were coated with murine S1EC at 10 µg/ml in PBS and incubated at 4°C overnight. Fibronectin was used as a positive control. Nonspecific binding sites were blocked with 0.1% BSA in PBS for 1 h at RT. HaCaT cells were seeded onto the slides at a density of 4×10^5^ cells/ml in serum free DMEM and incubated for 2 h at 37°C. The slides were washed with PBS containing 1 mM MgCl_2_ and 1 mM CaCl_2_, followed by a fixation of cells for 8 minutes in 2% paraformaldehyde in PBS at room temperature. The fixative was removed and the cells were washed with PBS. Permeabilization of the cells was achieved using PBS with 0.5% saponin, 2% horse serum and 2.5% methanol for 45 min at room temperature. The antibodies against vinculin or the β6 integrin were diluted in the same solution and incubated on the slides for 60 min at room temperature. The slides were then washed with blocking solution and the detection was performed using Cy-3 labeled anti-mouse IgG and Alexa 488-labeled goat anti-rabbit IgG. After final wash the samples were embedded in polyvinyl alcohol and images of cells were taken on Zeiss Axiophot fluorescence microscope.

## Results

### Identification of a Heparin-binding Motif

**Figure 1:**
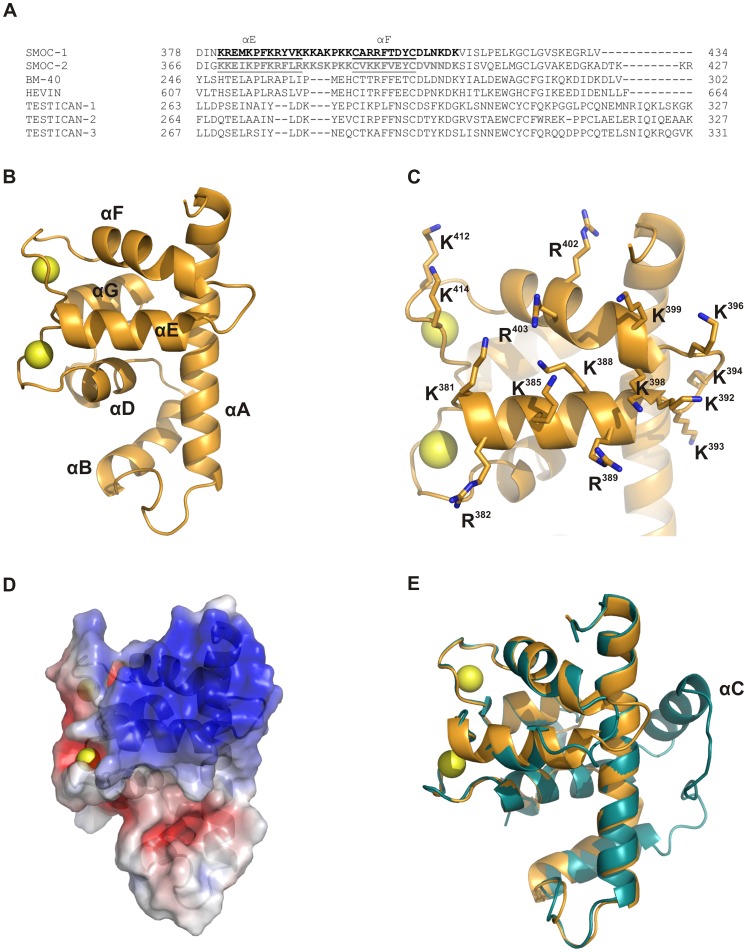
Identification of the heparin binding site in the primary and tertiary structure of S1EC. (**A**) Sequence alignment of the part of the EC domain which contains the potential glycosaminoglycan-binding site with other members of BM-40 family. The putative GAG-binding motive is shown in bold, black for SMOC-1 and grey for SMOC-2, and the positions of α-helices E and F are underlined. (**B**) Homology model of the S1EC domain in cartoon representation. Helices are numbered according to the BM-40 EC domain [10] and calcium ions in EF hands are represented as yellow spheres. (**C**) The putative heparin-binding region in helices E and F. Positively charged residues are shown as sticks. (**D**) Surface potential of the S1EC domain calculated with APBS Software. (**E**) Superposition of the S1EC model (shown in orange) and the BM-40 EC domain (shown in blue). All images were prepared with PyMOL (DeLano Scientific; http://www.pymol.org).

The alignment of the primary structure of the SMOC EC domains with other members of the BM-40 family revealed a cluster of positively charged residues unique to the SMOCs (Figure 1A). This region was mapped to the E and F helices of the domain (according to the terminology proposed in [10]), the loop connecting both helices and the second EF hand. A homology model of the S1EC domain was calculated based on the crystal structure of the BM-40 EC domain and is shown in Figure 1B. The positively charged residues in helices E and F (Figure 1C, shown as sticks) form a large interaction surface with a strong positive potential (Figure 1D). The overall comparison of the model with the crystal structure of the BM-40 EC domain shows that apart from the minor insertion between helices E and F, the major structural difference between both is the absence of helix C in the SMOC EC domain (Figure 1E), which is conserved in both SMOC paralogs.

### Size-exclusion Chromatography (SEC)

**Figure 2:**
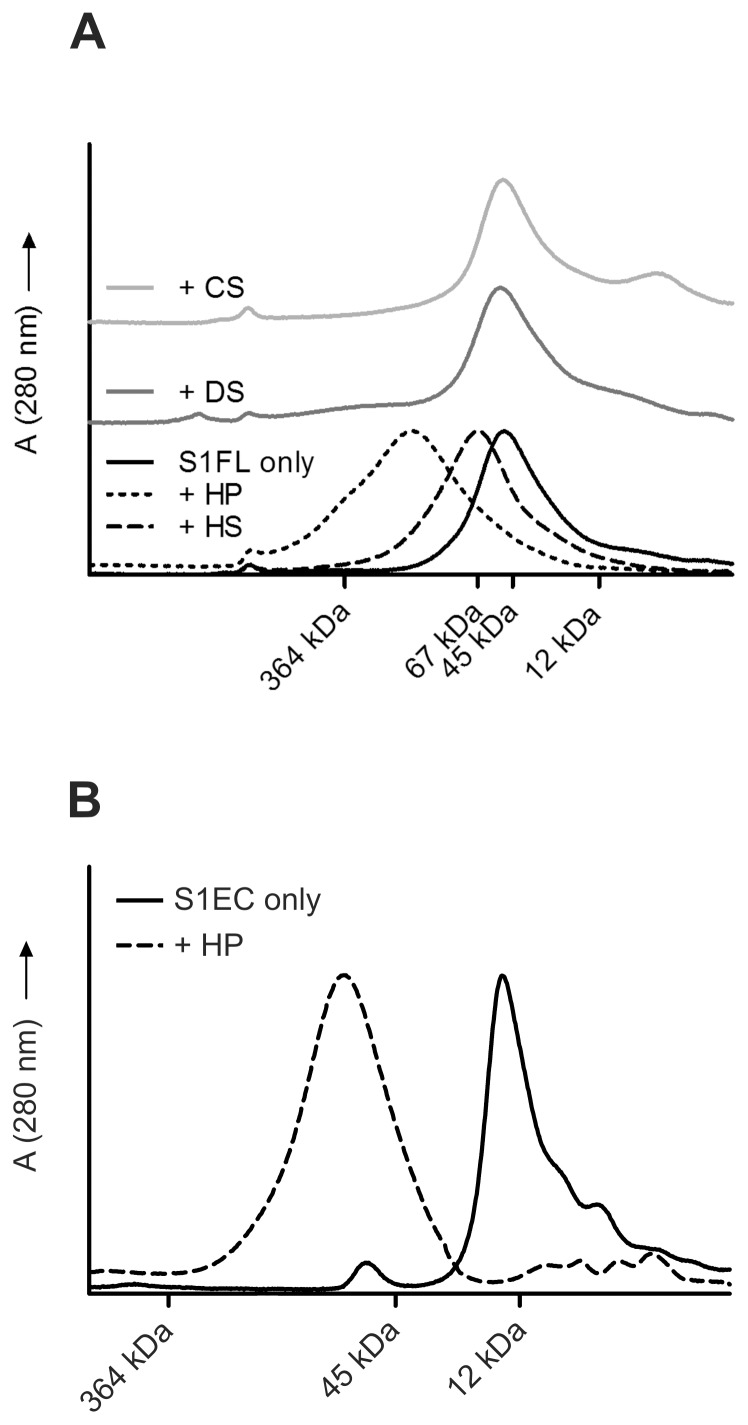
Size-exclusion chromatography of SMOC-1 and S1EC in the presence and absence of glycosaminoglycans (GAGs). (**A**) Elution profiles of S1FL under different conditions. The GAGs used were heparin (HP), heparin sulfate (HS), chondroitin sulfate (CS) and dermatan sulfate (DS). All runs were performed in 20 mM sodium phosphate buffer pH 7.4 containing 150 mM NaCl. (**B**) Elution profile of S1EC domain from a Superdex 200 SEC column in the presence and absence of heparin (HP). Both runs were performed in 20 mM sodium phosphate buffer pH 7.4 containing 150 mM NaCl. Elution volumes of standard proteins (Dalton Standards MS-II) are given in both diagrams.

Binding of S1FL to different GAGs was experimentally tested by analytical SEC in the presence of GAGs based on the assumption that the putative SMOC-1/GAG complexes will elute from the column earlier than the SMOC-1 alone due to higher molecular masses of the complexes. The experiments were performed with four different sulfated GAGs: HS, HP, CS and DS (See the Materials section for the molecular masses of the GAGs). As shown in Figure 2A, SMOC-1 alone eluted from the column at its expected size of 45 kDa. In the presence of HP SMOC-1 eluted in a broad peak shifted towards higher molecular masses, indicating complex formation. As expected, the SMOC-1/HP interaction was predominantly electrostatic and was readily abolished by increased salt concentration (not shown). In the presence of HS, the SMOC-1 peak was shifted to a lower elution volume with a mean molecular mass of approximately 60 kDa indicating a 1∶1 SMOC-1:HS chain binding ratio. Addition of DS or CS caused no changes in the elution profile of SMOC-1, indicating that there was no interaction between these GAGs and SMOC-1. Given these results, we can predict that SMOC-1 binding is specific for HS and HP and that a high degree of sulfation is necessary for the interaction. For this reason all further experiments were performed only with HP, the results, however, can also be extended to HS. Since the proposed binding site is located in the EC domain, we have repeated the experiments with the recombinant S1EC domain. As shown in Figure 2B the S1EC domain did indeed bind HP.

### CD Spectroscopy

**Figure 3:**
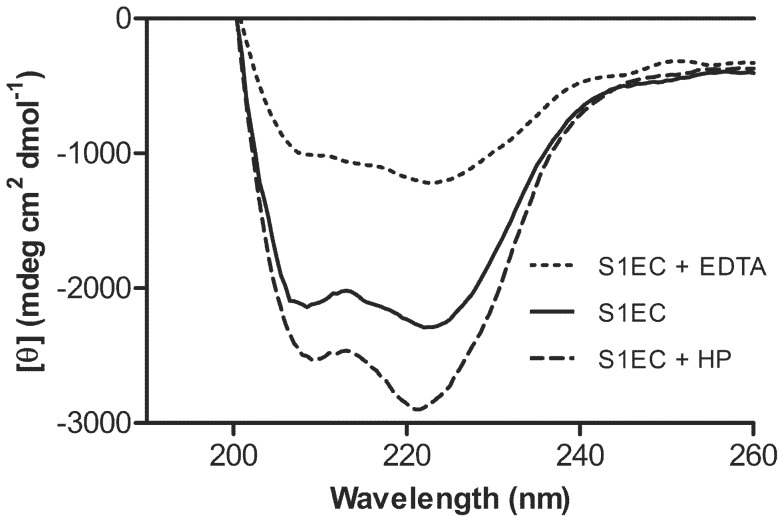
Circular dichroism spectra of S1EC. Spectra were recorded using a protein concentration of 5 µM in 20 mM HEPES containing 50 mM NaCl and 2 mM CaCl_2._ The solid line represents S1EC in the presence of 2 mM calcium ions, the dotted line represents S1EC in the presence of 5 mM EDTA and the dashed line S1EC in the presence of 50 µM heparin.

The CD spectra of recombinant S1FL have been published previously [8], [16]. Here we used circular dichroism to investigate the folding state and secondary structure content of the recombinant SMOC-1 EC domain, to verify its ability to bind calcium ions and to determine whether heparin affects the folding state of the domain. CD spectra of S1EC in the presence of calcium ions showed two negative elliptical peaks at 210 and 222 nm, characteristic for proteins with predominantly α-helical structure, indicating the correct folding of recombinantly expressed S1EC (Figure 3). Addition of EDTA, chelating Ca^2+^ ions in EF hands, reduced the ellipticity and decreased the ratio between molar ellipticities at 222 and 210 nm, suggesting a decrease in *α*-helical content [25]. Addition of heparin resulted in an increase in signal intensity and further increase in the ratio between molar ellipticities at 222 and 210 nm. The same effect was observed when heparin was added to the EC domain in the presence of EDTA (data not shown) although the intensity of the negative ellipticity was weaker than in the presence of calcium.

### Intrinsic Tryptophan Fluorescence

**Figure 4:**
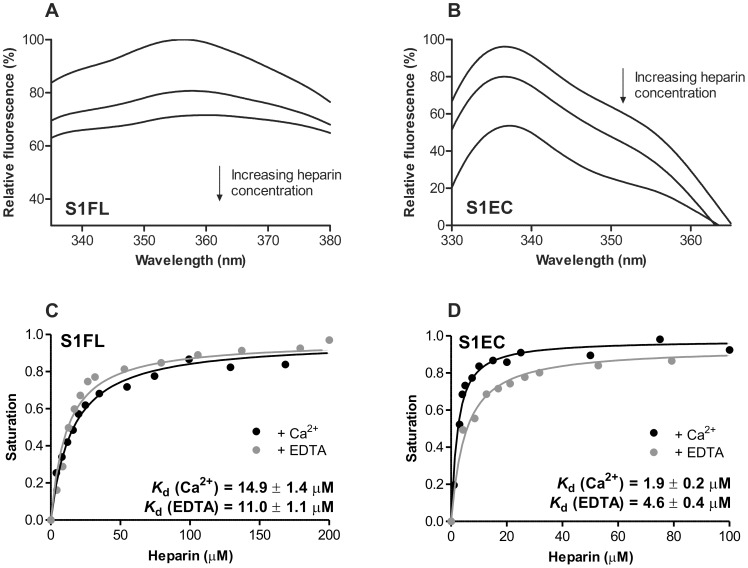
Intrinsic tryptophan fluorescence of SMOC-1 and S1EC in the presence of heparin. (**A, B**) Changes in intrinsic fluorescence emission spectra of SMOC-1 and S1EC, respectively, in the presence of various concentrations of heparin. Proteins (5 µM) were incubated with increasing amounts of heparin (0–200 µM, top line to bottom line, respectively). An excitation wavelength of 295 nm was used and spectra recorded from 300 to 500 nm. The observed relative changes in intrinsic fluorescence were plotted as a function of heparin concentration and transformed to degree of saturation. (**C, D**) Plots of fluorescence intensity versus heparin concentration. The solid black lines represent the best-fit curves calculated with Equation 2. Calcium binding of SMOC-1 or S1EC to heparin was also assessed in the presence of 5 mM EDTA. Best-fit curves are shown as grey solid lines. All calculated *K*_d_ values are given in the plots. The analysis was performed with GraphPad Prism 5.0 Software.

SMOC-1 contains four Trp residues; two of them are in the TY domain, one in the domain unique only to SMOC proteins and one in the EC domain. This enabled us to study the binding of heparin to the S1FL as well as the S1EC domain by intrinsic tryptophan fluorescence spectroscopy. The emission spectrum of SMOC-1 had a maximum at 357 nm. Addition of heparin resulted in decreased fluorescence intensity and a slight shift of the maximum towards higher wavelengths (Figure 4A). The S1EC domain had a broad emission spectrum with a peak at 338 nm. Binding of heparin strongly reduced the fluorescence intensity but did not affect the shape of the spectrum (Figure 4B). Changes in fluorescence at maximum intensity were plotted versus the concentration of heparin. Binding of heparin to SMOC-1 and S1EC produced a hyperbolic curve consistent with one-site binding of ligand to the receptor. Fitting Equation 2 to the experimental data, we calculated an equilibrium dissociation constant *K*_d_ of 14.9±1.4 µM for the binding of heparin to SMOC-1 (Figure 4C) and 1.9±0.2 µM for S1EC (Figure 4D). Addition of EDTA had little effect on heparin binding to either of the recombinant proteins and the binding constants remained virtually identical. This shows that binding of heparin is independent of calcium ions and is in agreement with data obtained using CD spectroscopy.

### Adhesion of HaCaT Cells to SMOC-1

**Figure 5:**
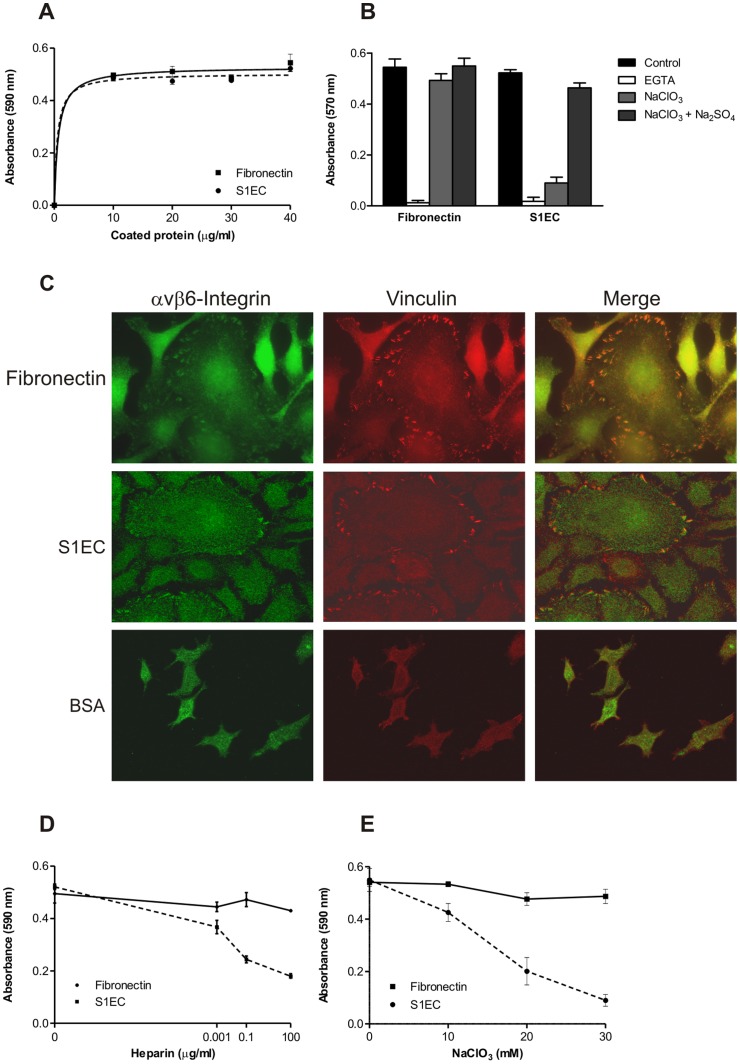
Adhesion of HaCaT cells to S1EC. (**A**) HaCaT cells (1 × 10^6^ cells/ml) were plated on microtiter wells coated with the indicated amount of S1EC or fibronectin that served as a positive control. Attached cells were stained with cresyl violet and the extracted dye was quantified by absorbance at 590 nm. (**B**) Bars represent HaCaT cells with 5 mM EGTA in serum-free DMEM media as well as HaCaT cells grown in low-sulfate medium containing 30 mM chlorate without or with addition of 10 mM Na_2_SO_4_. All cells were preincubated with reagents at 37°C for 30 min and then plated on S1EC (5 mg/ml) or fibronectin (1 mg/ml). Data shown are mean ± S.D. of three determinations and are representative of at least three experiments. (**C**) HaCaT cells grown on either human fibronectin or murine S1EC were stained with a rabbit anti-β6 integrin antibody (green) or with a mouse anti-vinculin antibody (red) to show the recruitment of the integrin to focal adhesions. In the right-most panels the superposition of the two stainings is shown. (**D**) HaCaT cells were plated on microtiter wells coated with S1EC (5 mg/ml) or fibronectin (1 mg/ml). Increasing amounts of heparin were added to the cells prior to plating. (**E**) HaCaT cells were cultured in low sulfate medium containing the indicated amount of sodium chlorate for 24 h, washed, harvested, and then plated on S1EC (5 mg/ml) or fibronectin (1 mg/ml).

It has been shown previously that the SMOC-2 EC domain supports human HaCaT keratinocyte adhesion [13]. To determine whether S1EC has a similar activity and to determine the role of heparin binding, we have tested the adhesion of HaCaT cells on immobilized S1EC under different conditions. HaCaT cells adhered to S1EC in a dose-dependent and saturable manner similar to fibronectin which was used as a positive control (Figure 5A). As expected, addition of EGTA to the culture medium completely blocked epithelial cell adhesion (Figure 5B), indicating the involvement of calcium-dependent cell adhesion receptors such as integrins. Indeed, the αvβ6 integrin receptor has been identified as the receptor responsible for HaCaT adhesion to the SMOC-2 EC domain [13]. To identify the locations of receptors on the surface of S1EC-adhered HaCaT cells and to confirm the presence of focal adhesions, colocalization of the ß6 integrin with vinculin, a focal adhesion marker, was examined (Figure 5C). Fluorescent staining against the ß6 integrin revealed its presence in vinculin-positive regions, confirming its involvement in S1EC-mediated HaCaT adhesion. Moreover, adhesion of HaCaT cells to S1EC was comparable to the staining in the corresponding positive control. Since it is known that in epithelial cells ß6 subunit pairs only with αv subunit [26], it can be concluded that the binding of HaCaT cells to S1EC is probably mediated by the αvß6 integrin receptor.

To determine the involvement of the heparin-binding activity of S1EC in cell attachment, various amounts of soluble heparin were added to the cells prior to coating. Remarkably, as little as 1 ng/ml heparin was sufficient to elicit a detectable inhibition, whereas 100 µg/ml showed pronounced disability for HaCaT binding to S1EC (Figure 5D). In comparison, the addition of the same amount of heparin had virtually no effect on HaCaT adhesion to fibronectin.

Since the production of heparin *in vivo* is restricted to mast cells, the likely biological targets for S1EC are heparan sulfates in the form of HSPGs. To evaluate the dependence of S1EC mediated HaCaT adhesion on cell surface GAGs, the cells were cultured in low sulfate RPMI-1640 media with 10% dialyzed FBS in the presence of sodium chlorate, an inhibitor of 3-phosphoadenosine 5′-phosphosulfate synthesis, to block sulfation of proteoglycans. As shown in Figure 5E, cells grown in the presence NaClO_3_ showed reduced adhesion to S1EC. The reduction was concentration dependent and reached 75% inhibition at 30 mM NaClO_3_. Inhibition of adhesion was specific and did not result from cytotoxicity of the chlorate, as adhesion on fibronectin was not affected by growth in chlorate. The inhibitory effect of sodium chlorate on cell adhesion to S1EC was reversed by the inclusion of 10 mM Na_2_SO_4_ in the culture medium, thus verifying that it is indeed the sulfation block that results in this inhibitory effect (Figure 5E).

### Heparin Binding and Adhesive Properties of Heparin-binding Impaired Mutants

**Figure 6:**
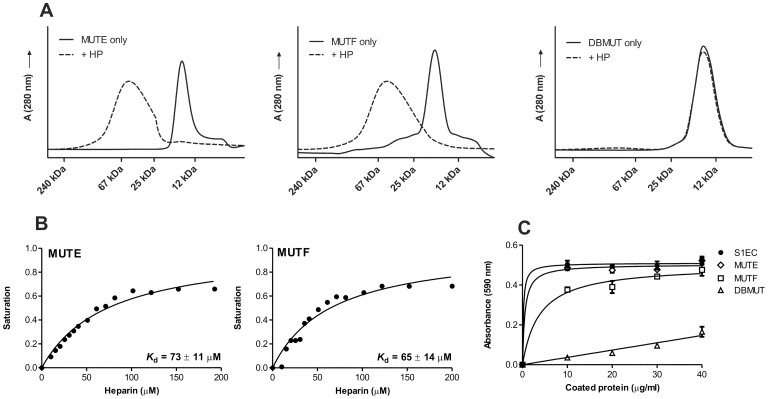
Heparin binding and adhesive properties of heparin-binding impaired mutants. (**A**) Elution profiles of MUTE, MUTF and DBMUT from a Superdex 200 SEC column in the presence and absence of heparin. All runs were performed in 20 mM sodium phosphate buffer pH 7.4 containing 150 mM NaCl. Elution volumes of standard proteins (Dalton Standards MS-II) are given in the diagrams. (**B**) Changes in intrinsic fluorescence emission spectra of MUTE and MUTF, respectively, exposed to various concentrations of heparin. Proteins (5 µM) were incubated with increasing amounts of heparin (0 to 200 µM). The solid black line represents the best-fit curves calculated by use of Equation 2, which was used to derive *K*_d_ values for MUTE and MUTF interactions with heparin. (**C**) HaCaT cells were plated on wells coated with the indicated amounts of wild-type S1EC as a control or mutant proteins MUTE, MUTF or DBMUT. Adhesion assay was performed as described before.

Although cell surface HSs are essential for S1EC-mediated adhesion of HaCaT cells, whether S1EC must interact with HSPGs through its predicted heparin-binding activity remained to be determined. To address this question, mutant S1EC proteins, impaired in heparin binding were prepared. The proposed binding site includes helices E and F of the domain. We have chosen four positively charged residues in each helix that were replaced by alanine residues by site-directed mutagenesis (Figure 1C), resulting in three mutant S1EC variants: variant MUTE with mutations in helix E (K381A/K385A/R389A/K392A), variant MUTF with mutations in helix F (K398A/K399A/R402A/R403A) and variant DBMUT with mutations in both helices. The SEC elution diagram in Figure 6A shows that MUTE and MUTF were still able to bind heparin. The addition of heparin resulted in appearance of broad peaks shifted towards higher molecular masses. No such difference between elution diagrams with and without heparin was seen for DBMUT (Figure 6A) indicating that this mutant did not bind heparin. To quantify the heparin-binding affinities of the mutants, intrinsic tryptophan fluorescence measurements were performed as described for SMOC-1 and S1EC. Both spectra were similar in shape to S1EC, having a maximum at 338 nm. Addition of heparin to MUTE or MUTF caused a decrease in fluorescence intensity but had no effect on the shape of the emission spectra (not shown). Plots of the difference in fluorescence intensity versus heparin concentration showed that MUTE and MUTF had a reduced affinity for heparin binding in comparison to S1EC (Figure 6B). The calculated dissociation constants for MUTE and MUTF were 73±11 µM and 65±14 µM, respectively. Intrinsic fluorescence measurements were also performed with DBMUT, which did not bind heparin on SEC. In this case, addition of heparin had no effect on the fluorescence emission spectra (not shown). On one hand this proves that reduction of Trp fluorescence intensity is indeed due to specific binding of heparin to the protein and on the other hand it confirms that DBMUT does not bind heparin. To assess the adhesive properties of mutants, varying concentrations of MUTE, MUTF or DBMUT were coated onto microtiter wells, and HaCaT cells were allowed to attach (Figure 6C). MUTE was able to support HaCaT adhesion in a manner similar to S1EC, while MUTF showed decreased binding even at higher concentrations. By contrast, DBMUT showed an obvious decrease in HaCaT adhesion, suggesting that S1EC-mediated adhesion of HaCaT cells is dependent on its binding to cell surface HSPGs and requires availability of both helices.

### Modeling, Docking

**Figure 7:**
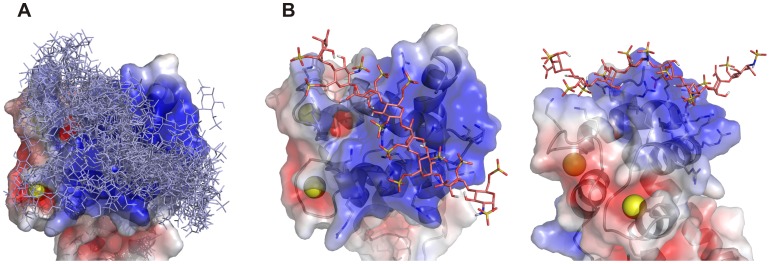
Docking of heparin octasaccharides to the proposed binding site on the S1EC. (**A**) Ensemble of all docking solutions that are in agreement with experimental data. The largest cluster of docking solutions is running across both helices and over the loop of the second EF hand (from the lower right to the upper left). (**B**) A selected docking solution from the largest cluster of docking orientations in two different perspectives. Heparin chains are shown as sticks. The surface potential was calculated with APBS software. Images were created with PyMOL (DeLano Scientific; http://www.pymol.org).

To gain additional insight into the heparin-S1EC interaction at the molecular level, we constructed computational models of the S1EC/heparin complex by docking heparin fragments to the proposed binding site *in silico*. Initial docking trials showed that the proposed binding site is sufficiently large to interact with at least six monosaccharide moieties of heparin. The results presented here were calculated with heparin octasaccharides. From the ensemble of docking solutions produced as described in the Experimental section, the solutions in agreement with experimental data were chosen. The main criterion for selection was that the GAG chain should interact with residues from both helices E and F for maximal affinity to be observed. The ensemble of all docking solutions (Figure 7A) shows that there are multiple possible binding modes for the heparin chain, however one orientation of the chain is preferred and represents the majority of all calculated solutions. An example model from this group of solutions was selected and is shown in Figure 7B. The heparin chain runs across both helices, roughly parallel to helix F and over the loop of the second EF-hand, interacting with at least eight residues from the receptor molecule along the interaction surface. Taking into account that the docking was performed with a rigid receptor molecule, the actual number of interacting receptor residues may indeed be higher.

## Discussion

SMOC-1 and SMOC-2 proteins are the most recently characterized members of the BM-40 family. Both of them are present in many cell types and are highly expressed during embryogenesis, wound healing, and other physiological processes involving extensive tissue remodeling [7]. To identify the molecular mechanisms behind these processes we focused on SMOC-1 protein and especially on its EC domain. Based on the high sequence homology between the heparin-binding regions of SMOC-1 and SMOC-2 (See Figure 1A), all results presented here for SMOC-1 can likely be extended to SMOC-2. As already shown for EC domains of other BM-40 family proteins, BM-40 [10], hevin [27] and testican [28] the EC module of SMOC-1 is an autonomously folding domain with a distinct affinity for calcium ions. A decrease in the negative molar ellipticity was observed when calcium ions were removed by adding EDTA. This demonstrates that the EF hands of S1EC are active in calcium binding, which is in agreement with previously published data on SMOCs [8], [16].

The EC domain of SMOCs contains a unique cluster of basic amino acids, representing a potential GAG-binding site that does not appear in other members of the BM-40 family. Our experiments have shown that S1FL as well as S1EC bound HP and HS but not CS or DS. The selectivity for heparin can be explained by the fact that heparin, on behalf of its dense sulfation, carries the amount of negative charges sufficient for the formation of ionic bonds to positively charged amino groups of the protein. It can be therefore seen that it is the lack of negative charge in the case of CS and DS that is causing repulsive forces between the sugar moiety and the protein and thus disabling energetically favorable binding. The likely biological binding partners of the SMOCs are HSs, which exhibit diverse modification patterns [29] and multiple “domains” with distinctive sulfation degrees are often present within a single chain. Our results indicate that SMOC-1 binds only to highly sulfated regions of HS which explains the lower stoichiometric ratio of S1FL binding to HS, resulting in higher elution volumes in comparison to the S1FL-HP complex. The data obtained using SEC where S1EC alone eluted at 15 kDa and S1EC in complex with heparin eluted as a broad peak with a maximum at approximately 75 kDa suggest that on average four EC domains can bind to a single heparin molecule. Taking into account the average disaccharide molecular mass of 570 Da, it follows that each EC molecule occupies six disaccharide units. This complies well with the results from molecular modeling where four disaccharide units were necessary to cover the complete binding site. The difference of two disaccharide unites can likely be attributed to hindrance between adjacent EC domains. As in the case of many other ECM proteins [30], [31], S1EC bound heparin in a calcium-independent manner, since chelation of the Ca^2+^ ions in EF hands did not result in a pronounced decrease in heparin-binding affinity. This indicates that correct conformation of helices involved in Ca^2+^-binding is not required for successful heparin binding.

At the sequence level, the proposed heparin-binding site on S1EC is a linear motif of about 20 residues in length which forms two alpha-helices running antiparallel to each other. Based on this, the motif can be described as a blend of a strictly linear motif and a higher order spatial motif. The large basic interacting surface is therefore comprised of two linearly contiguous clusters brought together through correct folding. In comparison to the recently identified heparin-binding site in transglutaminase-2 [32], [33], where two clusters of positively charged residues are 300 residues apart in the primary sequence, the basic clusters in S1EC are not distant at the sequence level but correct folding is still necessary for the formation of a functional heparin-binding site. The proposed heparin-binding site in S1EC contains two “CPC clip motifs” comprising one polar and two cationic residues, which has recently been identified as a common heparin-binding motif [34]. In addition, the S1EC-binding site also contains non-basic amino acids, known to be involved in heparin binding in other proteins: helix E contains a Glu residue, an amino acid that is supposed to be important for both acidic and bFGF to interact with heparin [35] as well as Tyr which may support heparin binding via formation of a hydrogen bond to hydroxyl groups on the heparin [36].

Great importance is lately attributed to HP and HS-binding proteins. It has been shown that HS can interact with a wide range of proteins due to its high content of charged groups and is essential in various biological processes due to its structural diversity [37]. Since HS, and not HP, is present in ECM, we investigated the physiological significance of S1EC binding by determining the role of S1EC in supporting the adhesion to epithelial cells. This property was recently described for SMOC-2 EC (S2EC) domain and integrins αvβ1 and αvβ6 were identified as the receptors involved in S2EC-mediated cell adhesion [13]. Since HaCaT cells exhibit the same calcium-dependent behavior in adhesion to S1EC, it seems reasonable to assume that these receptors are also involved in SMOC-1-mediated cell adhesion. Indeed, colocalization experiments using S1EC have identified integrin β6 at the sites of focal adhesions (see Figure 5C). Interestingly, formation of focal adhesions was not observed in other members of BM-40 family. Conversely, abrogation of focal adhesions occurred in the presence of BM-40 as well as hevin/SC1 [38], [39].

Since it is known that HSPGs can function as co-receptors in integrin-mediated cell adhesion, we have investigated whether cell adhesion on S1EC depends on its heparin-binding activity. Data acquired using several approaches indicated that the heparin-binding site must be available and functional to interact with cell-surface HS. Firstly, addition of soluble heparin reduced adhesion of HaCaT cells to S1EC in a dose dependent manner, ruling out the possibility that it is the conformational change caused by heparin binding that accounts for successful binding to integrin as recently shown for focal adhesion kinase [40]. The value of IC_50_ for heparin, estimated from cell adhesion assays is similar to the *K*_d_ value, calculated from intrinsic fluorescence measurements. Secondly, damaging the integrity of cell surface HSs by addition of sodium chlorate, which inhibits sulfation of GAGs, resulted in abrogated adhesion, and thirdly, the DBMUT showed reduced HaCaT adhesion activity. These data collectively suggest that it is the interaction with cell surface HSPGs that is a major factor for S1EC-mediated cell adhesion. The most likely targets are proteoglycans of the syndecan or glypican families. Especially the former are well known to play important roles as co-receptors in integrin-mediated cell adhesion [41]. However, we can conclude that HSPGs on the cell surface do not suffice for successful binding, since under chelating conditions HaCaT cells failed to adhere to S1EC even though recombinant S1EC retains its heparin-binding activity under these conditions. HSPGs can thus be considered as co-receptors, which stabilize the formed adhesion complex. While the interaction between S1EC and HSPGs alone may not be tight enough to promote cell adhesion, it is likely sufficient to serve a regulatory purpose. This hypothesis is further supported by the fact that S1EC retained a certain degree of cell-binding activity even in the presence of saturating concentrations of heparin (Figure 5D) and when the heparin-binding site was completely removed (Figure 6C).

Comparison with other known heparin-binding proteins shows that the interaction is of intermediate affinity, which supports the regulatory or accessory role of these interactions in cell adhesion. The affinity of SMOC-1 for heparin was measured using soluble heparin. The heparan sulfate found in the pericellular space is bound to the protein backbone which is in turn bound to the cell membrane. For this reason the heparan sulfate is not freely diffusible and its activity is not directly comparable to the activity of soluble heparin (the activity of pure solids is 1). Moreover, multiple spatially adjacent HSPG molecules can create the effect of a very high local “concentration” of heparan sulfate. Therefore, measurements with soluble heparin performed in dilute solutions are only a measure to compare the affinity of SMOC-1 for heparin to other known heparin-binding proteins and are not truly representative of *in vivo* situations. These are more closely simulated by the cell adhesion experiments which we have performed and which have clearly shown that the amounts of HSPGs on the cell surface are certainly sufficient to influence cell adhesion to SMOC-1.

Several HSPGs, including collagen XVIII, perlecan and agrin [20], are also abundant in the BM, a complex form of ECM, where SMOC-1 was shown to localize. Based on currently available data it is yet impossible to predict which of these HSPGs SMOC-1 can interact with. On the other hand not enough data is yet available to exclude the possibility of SMOC-1 binding to other proteins where it could fine-tune the physiological processes as an adaptor protein. Further information at the molecular level is therefore necessary to assess the role of SMOC-1 in ECM.

## Supporting Information

Figure S1 **SDS–PAGE analyses of recombinant S1EC, its mutants (MUTE, MUTF, DBMUT) and S1FL.** Analysis of the final preparations under non-reducing (N/R) and reducing (R) conditions. EC domain samples were run on a 15% polyacrylamide gel and S1FL was run on a 12% polyacrylamide gel. All proteins were stained with Coomassie Brilliant Blue R-250. Positions of calibrating proteins are given in kDa.
(TIF)

Methods S1 **Cloning of the constructs.**
(DOCX)
