# Supplementary figures and images for: The Heparin-Binding Activity of Secreted Modular Calcium-Binding Protein 1 (SMOC-1) Modulates Its Cell Adhesion Properties
Source: PLoS One. 2013 Feb 21;8(2):e56839. doi: 10.1371/journal.pone.0056839 (PMC3578922; doi:10.1371/journal.pone.0056839)

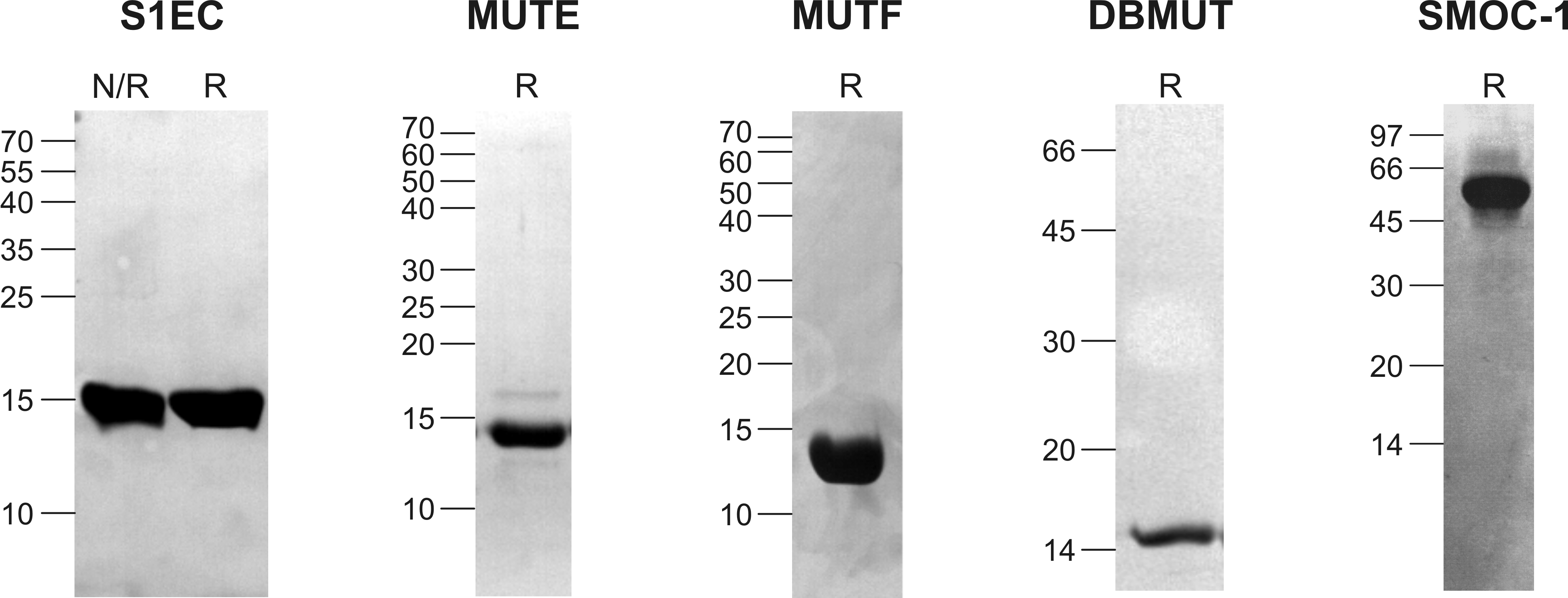

Supplement: Figure S1 — SDS–PAGE analyses of recombinant S1EC, its mutants (MUTE, MUTF, DBMUT) and S1FL. Analysis of the final preparations under non-reducing (N/R) and reducing (R) conditions. EC domain samples were run on a 15% polyacrylamide gel and S1FL was run on a 12% polyacrylamide gel. All proteins were stained with Coomassie Brilliant Blue R-250. Positions of calibrating proteins are given in kDa. (TIF) [file pone.0056839.s001.tif]
