# Supplementary material for: The Heparin-Binding Activity of Secreted Modular Calcium-Binding Protein 1 (SMOC-1) Modulates Its Cell Adhesion Properties
Source: PLoS One. 2013 Feb 21;8(2):e56839. doi: 10.1371/journal.pone.0056839 (PMC3578922; doi:10.1371/journal.pone.0056839)
Supplement: Methods S1 — Cloning of the constructs. (DOCX) [file pone.0056839.s002.docx]

**Methods S1**

**Cloning of the constructs­­**

For construction of mouse wild type extracellular calcium-binding domain (mS1EC) following oligonucleotides were used as primers:

mS1EC forw: 5’-GCCCGCTAGCGAGAGTGACGCCAGAGCC-3'

mS1EC rev: 5’-CAATGACTGCGGCCGCTTAGACAAGGCGTCCGATGAAGC-3'

Construction of human full-length SMOC-1, wild type extracellular calcium-binding domain (S1EC) or three S1EC mutants (MUTE, MUTF and DBMUT) were produced using the following oligonucleotides as primers:

S1FL forw: (1) 5'-CATGCCATGGCCACCGCACCACAGG-3'
S1FL rev: (2) 5'-CCGCTCGAGGACGAGGCGTCCTTCTTTGC-3'
S1EC forw: (3) 5'-CATGCCATGGCCCTACCAGGCTGTCCA-3'
MUT1 rev: (4) 5'- AGCCTTGAAGGGAGCCATCTCCCGAGCGTTAATGTCGTTGCT -3'
MUT2 forw: (5) 5'- GCTCGGGAGATGGCTCCCTTCAAGGCTTACGTGGCTAAGAAAGCCAAGCCC -3'
S1EC rev: (6) 5'- CATGCTCGAGGACGAGGCGTCCTAC -3'

S1FL and S1EC were produced by the PCR using the combination of oligonucleotides 1 and 2 or 3 and 6 as primers, respectively. S1EC mutants harboring alanine substitutions within the predicted putative heparin-binding motif between amino acids 281–292 and 398–403 were constructed by site-directed mutagenesis using a two-step PCR procedure. For the MUTE mutant the internal reverse primer 4 and for the MUTF mutant internal forward primer 5 were used. The outside primers used in the PCR for both mutants were 3 and 6, respectively. To construct the double mutant (DBMUT), the MUTE mutant cDNA was used as template, and PCR mutagenesis was performed using internal primers for MUTF mutant. The full-length human SMOC-1 cDNA clone was used as template in all amplifications. Resulting products were gel-purified, combined, and used as template for the second step PCR using the outside primers 3 and 6.
